# Challenges and Learning Needs for Providers of Advanced Cancer Care: Focus Group Interviews with Physicians and Nurses

**DOI:** 10.1089/pmr.2020.0059

**Published:** 2020-09-30

**Authors:** Tonje Lundeby, Torunn Elin Wester, Jon Håvard Loge, Stein Kaasa, Nina Kathrine Aass, Kjersti Støen Grotmol, Arnstein Finset

**Affiliations:** ^1^ Regional Advisory Unit in Palliative Care, Department of Oncology, Oslo University Hospital, Oslo, Norway.; ^2^ European Palliative Care Research Centre (PRC), Department of Oncology, Oslo University Hospital and Institute of Clinical Medicine, University of Oslo, Oslo, Norway.; ^3^ Institute of Clinical Medicine, University of Oslo, Oslo, Norway.; ^4^ Department of Behavioural Sciences in Medicine, University of Oslo, Oslo, Norway.; ^5^ Norwegian National Unit for Rehabilitation for Rheumatic Patients with Special Needs, Oslo, Norway.

**Keywords:** communication, integration, oncology, palliative care, qualitative study

## Abstract

***Background:*** Implementation of integrated oncology and palliative care improves patient outcomes but may represent a demanding task for health care providers (HCPs).

***Objective:*** To explore physicians' and nurses' perceived challenges and learning needs in their care for patients with advanced cancer, and to analyze how these perceptions can provide insight on how to improve care for patients with advanced cancer in an integrated care model.

***Methods:*** Residents in oncology, oncologists, nurses, and palliative care physicians were recruited to participate in focus group interviews. Six focus group interviews were conducted with 35 informants. Data were analyzed according to principles of thematic analysis.

***Results:*** The discussions in the interviews concerned three broad themes: an emphasis on patients' best interest, perceived as hindered by two sets of barriers; unsatisfactory organizational conditions such as time pressure, lack of referral routines, and few arenas for interdisciplinary collaboration, was perceived as one barrier. The other barrier was related to the appraisal of other HCPs' clinical practices. Participating HCPs expressed in general a positive self-view, but were more critical of other HCPs.

***Conclusion:*** Currently, implementation of measures to improve care for patients with advanced cancer appears to be challenging due to cultural and organizational factors, and how HCPs perceive themselves and other HCPs. HCPs' perception of challenges in patient care as not related to themselves (externalization) might be an essential obstacle. Interventions targeting both HCP-related and organizational factors are needed. Particularly important are measures aimed at reducing fragmentation and improving collaboration in care.

## Introduction

In Norway there is a well-functioning public health care system that provides high-quality care, including cancer care. According to a 2015 comparison of the Organisation for Economic Cooperation and Development, survival in Norway is above average for different types of cancer and is among the best worldwide on breast and cervical cancer.^1^

However, highly specialized systems may also give rise to fragmented care.^2,3^ Norwegian patients and health care providers (HCPs) within oncology confirm this and report care as uncoordinated.^4,5^ Patients also experience lack of continuity in care providers and care delivery, and they report not being sufficiently involved in decisions about themselves.^6^

To overcome the challenges and consequences of fragmented care, the Norwegian government established in 2015 28 specified patient care pathways in cancer care aiming to improve interprofessional collaboration and coordination at different levels of the health care system.^7^ Based on these pathways, plans tailored for the individual patient were to be organized with specific emphasis on his or her values and preferences. The patient care pathways have been a success with regard to shortened time from referral to treatment onset, that is, better coordination in the early phases of the cancer trajectory. However, the individualization and specifics regarding how and when to integrate palliative care in the treatment plan for patients with advanced cancer still need improvement.^8^

The complexity of the palliative phase that places high demands on patient care has been emphasized in Official Norwegian Reports since 1984 (Ref.^9^). However, today, ∼40 years later, palliative care is still not offered to all patients with advanced cancer. Some patients are referred to palliative care at the cessation of tumor-directed treatment, whereas others are referred even closer to end of life. A large number of patients are not referred to palliative care at all.^10,11^ Thus, a specific structure for the advanced cancer trajectory, which at the same time is amenable to the individual patient, is needed. If implemented successfully, ample evidence now shows that cancer care coordinated in such pathways with early integration of palliative care provides improved patient outcomes compared with more fragmented care delivery.^12,13^

Early integration of oncology and palliative care has, however, proved challenging to implement due to several barriers.^14–16^ These barriers include doubts about the usefulness and importance of palliative care among oncologists, insufficient collaboration between various health care professions, noninvolvement of patients in decisions of care, the misunderstanding of palliative care as end-of-life care only, and insufficient organizational factors.^14,15,17–21^

Owing to these and potential other barriers, implementation of coordinated integrated care for patients with advanced cancer may represent a demanding task for HCPs. Better knowledge of the challenges HCPs perceive in their care for patients with advanced cancer and what learning needs they have can be informative to understand how to achieve this.

Our research questions addressed in this article are, therefore:

1. What challenges do HCPs perceive in their care for patients with advanced cancer?
2. What are the perceived educational needs of HCPs providing advanced cancer care?
3. How can these perceptions provide insight on how to improve advanced cancer care in a model integrating oncology and palliative care?

## Methods

The present focus group study was conducted as a part of a national cluster-randomized trial (ClinicalTrials.gov Identifier: NCT03088202) currently conducted in Norway called PALLiative care Integrated in ONcology (PALLiON). Implementation of care pathways with early integration of oncology and palliative care is the main element of the intervention in PALLiON in addition to increased focus on the patient's perspective, needs, and involvement in care decisions. The focus groups were conducted during preparation of the content of the intervention to ensure that it was relevant for HCPs.

### Design

This study applied a qualitative approach in which focus group interviews were conducted. Focus groups facilitate open discussions and sharing of opinions and experiences, providing a rich dataset.

### Setting and sample

Residents in oncology, oncologists, nurses, and palliative care physicians were recruited from the department of oncology at Oslo University Hospital, Norway, 1 of 12 oncological departments participating in PALLiON. The participants were recruited from two different campuses at the department using purposive sampling.

An invitation was sent to heads of palliative care and oncological sections at the department, who then chose which participants to invite. Variation in gender and age was pursued, yet this was a challenge since the majority of nurses and physicians are female. Newly hired clinicians (less than one year experience) were not recruited. Six focus group interviews with 35 informants were conducted (Table 1).

**Table 1.** Participant Description

Group number	Professional group	Number of participants	Gender (male/female)
1	Oncologists	5	2/3
2	Palliative care physicians	5	0/5
3	Residents in oncology	7	1/6
4	Palliative care nurses	6	0/6
5	Oncology nurses localization 1	6	0/6
6	Oncology nurses localization 2	6	0/6

### Interview guide

The interview guide included the following topics: challenges in treatment and care for patients with advanced cancer, interprofessional collaboration, patient involvement in decisions, and perceived individual learning needs.

### Data collection

The interviews were conducted by A.F., K.S.G., and T.L. alternating between being moderator or assistant. Each interview lasted approximately one hour (mean 61 minutes 30 seconds, range 53–67 minutes) and was audiotaped. After verbatim transcription of the interviews using HyperTranscribe, the data were imported to NVivo software for analysis.

### Data analysis

The transcribed text was analyzed using thematic analysis.^22^ Thematic analysis is a data-driven inductive approach, and involves the search for and identification of common threads that extend the interviews.^23^

First, all interviews were listened to and transcripts were read through several times independently by two of the authors (T.L. and T.E.W.). Second, initial codes and notes considered as pertinent features of the data were generated. The third stage involved searching for themes—overarching topics and patterns in the data. The identification of themes was made independently of the interview guide. To ensure confirmability, we chose to have two authors independently analyze the data. T.L. has a background from social psychology and clinical communication and T.E.W. is an oncology nurse with clinical, organizational, and leadership background from oncology and palliative care. T.L. and T.E.W. identified the same themes and patterns in the data. Both had noted two identical topics that we chose to pursue in further analysis, and that later on were divided into three themes. T.L. then categorized all statements in one interview into initial or new codes and developed a thematic map to aid generation of main themes. Then T.E.W. used the same thematic map and categorized all statements in the same interview. If statements did not fit into a code, a new code was created. T.E.W. and T.L. then went through all codes and statements together, to discuss agreement and to decide upon themes. T.L. coded the remaining five interviews. Themes and descriptions were discussed at length, both with each other and with the other coauthors.

The themes were further analyzed according to the conceptual framework of the article.

### Ethical considerations

All participants signed a consent form. When patients are not involved, a formal ethical approval is not asked for by the Regional Committees for Medical and Health Research Ethics in Norway.

## Results

The discussions in the interviews concerned three broad themes: (1) an emphasis on patients' best interest, perceived as hindered by two sets of barriers; (2) unsatisfactory organizational conditions; and (3) other HCPs' clinical practices.

### Emphasis on patients' best interest

The first theme is an ideal and a desire expressed by both physicians and nurses to provide best possible care. Participants emphasized elements of the patient-centered approach^24^ such as focusing on patients' values, quality of life, understanding, and needs. Information as a necessary prerequisite for patient involvement was highlighted. Physicians and nurses, both in oncology and palliative care, described their own clinical work as consistent with these values. They reported opening up for talking about difficult and emotional topics. They also described asking about the patients' preferences and actively involving them in decisions about care.

Oncologist (Focus group 1) about involving the patients in decisions:

*It is completely natural for me to include patients in decisions. I experience that most patients want to be involved. We for example, consider whether chemo should be given throughout the summer or not, and we arrange treatment breaks to make vacations possible.*

Palliative care physician (Focus group 2) about information provision and decisions:

*Some patients don't want to die, others are more concerned with pain relief. Some would rather experience pain than nausea, whilst others are the other way around. So, I take my time and explain to the patient that he or she has choices in this respect*.

Although there was an emphasis in all groups on patients' best interests, two sets of barriers were recognized and elaborated upon.

### Unsatisfactory organizational conditions

Factors concerning the organization of care were appraised as hindering optimal patient care. Sufficient time in consultations and continuity of care were considered by the participants as two essential elements, but were regarded as unsatisfactory in present practice. Both physicians and nurses reported that time pressure hindered exploration of the patient perspective and conversations about sensitive topics. Moreover, participants claimed that difficult conversations were postponed due to lack of continuity in the physician–patient relationship.

Oncologist (Focus group 1) about time in consultations:

*I also find that to be the most challenging; when you have twenty minutes to inform about disease progression, and the patient has never seen you before*.

Oncologist (Focus group 1) about the positive effect of physician–patient continuity:

*Sometimes I forget that when I know the patients, they know me too, and we end up in a dialogue.*

Referral practices represented another prominent challenge. Many of the participants agreed on the importance of early referral to palliative care, yet described the time point for referral as often being too late.

Palliative care physician (Focus group 2) about referral to palliative care:

*They are sometimes referred (to palliative care) when they are dying, and that is far too late. It's a long journey before you get there.*

Oncologist (Focus group 1) about own referral practices:

*I refer to the palliative care unit early. (…) Then they become familiar with the services, which in turn ease transfer of the patient when ending (tumor-directed) treatment.*

Oncologist (Focus group 1) about others' lack of referral to palliative care:

*I have seen patients who obviously have gone a long time without anyone noticing the lack of (palliative) support.*

The need for more organized collaboration between physicians and nurses and across subdivisions of oncology and palliative care was expressed in several of the groups. Both nurses and residents requested specific arenas for collaboration. They argued that increased collaboration would benefit the patients by, for example, improved information flow regarding symptoms and patient preferences. Especially nurses wanted to organize care in a way allowing them to contribute more.

Oncology nurse (Focus group 6) about collaboration and a wish to increase own contribution:

*Collaboration would have made the everyday life easier for the physicians as well—when making important decisions, decisions regarding treatment. To discuss that with someone else that knows the patients, from another view—I think that would be helpful. Therefore, I find it a pity they don't include us more.*

Oncology nurse (Focus group 6) about nurse–physician collaboration:

*I think the collaboration in general is pretty bad (…) it is basically non-existing. We really miss this dimension in our work.*

Views on and practices of collaboration varied across sections within the department. Although oncologists in general reported communication with other HCPs and the palliative care services as satisfying, most of the oncology nurses reported that physician–nurse communication was lacking. The nurses found it difficult to approach the physicians and remarked on the lack of arenas to meet.

Dialogue excerpt between several oncologists and the moderator (M) in Focus group 1, about collaboration with palliative care services:

*M: (…) is there a need for a more structured collaboration between palliative care and oncology?*

*Oncol. 1: Well, it works fine for me*

*Oncol. 2. I also think it works well*

*Oncol. 6: Yes, works well*

*Oncol. 3: They really want to contribute*

### Critical appraisal of other HCPs' clinical practices

Another set of barriers to succeed with optimal care that was identified by the participants was the clinical practice of other HCPs. Both nurses and physicians expressed, in general, a positive self-view but were more critical toward other HCPs' clinical practices. This was especially prominent regarding the topic of tumor-directed treatment.

Nurses, residents in oncology, and palliative care physicians described that a greater emphasis on tumor-directed treatment than on the whole patient was prevailing among many oncologists. These statements exposed a culture and an alleged willingness to provide tumor-directed treatment late in the disease trajectory and to very ill patients.

Oncology nurse (Focus group 5) about treatment late in the disease trajectory:

*They are way too ill when they come for chemo. And afterwards we read the obituaries—oh, that patient was here last week and now he's dead.*

Some residents and nurses stated that the tumor-directed treatment focus could lead to earlier death and lower quality of life.

Resident (Focus group 3) about futile treatment:

*I think we are killing people with the treatment we provide. Our information about the side effects of the treatment doesn't get through.*

Both nurses and residents had experienced chemotherapy being administered to dying patients.

Oncology nurse (Focus group 5) about a willingness to treat late in the disease trajectory:

*Old fragile patients get a considerable amount of chemotherapy and we ask ourselves; why are we doing this? It has been taken too far now, what are we doing?*

Neither nurses nor residents recognized excess focus on tumor as prevailing in their own professional group. They all pointed outward, to the oncologists. The oncologists did not discuss their own tumor-directed treatment practices.

Resident (Focus group 3) about consultants continuing treatment to fulfill hope:

*It's like we want to give the patients hope that we will continue to treat, treat, treat, treat.*

Resident (Focus group 3) about treatment decisions:

*It might be easier to continue than to quit tumor-directed treatment since you then have something to offer, although it might not be the best option for the patient.*

The emphasis on tumor-directed treatment was perceived by some participants to be at the expense of required symptom treatment. Insufficient focus upon psychosocial issues, pain, and other bothersome symptoms was described as a challenge.

Palliative care nurse (Focus group 4) about symptoms going untreated:

*We see many patients with a long history of high symptom burden who are not being referred to us.*

Although residents expressed some of their insecurities and shortcomings, nurses, oncologists, and palliative care physicians expressed confidence in their own skills. Accordingly, neither physicians nor nurses reported urgent learning needs. They did, however, point out some areas they perceived as “more challenging than others.” Young patients, patients with small children, angry patients, and cultural differences were reported as the most challenging when asked about perceived learning needs.

## Discussion

In our thematic analysis of data from six focus groups, we identified three topical areas of barriers and educational needs to achieve coordinated and integrated care for patients with advanced cancer.

First, independent of their profession, HCPs strongly emphasized the patients' best interest to guide care and treatment. Focus on patient values and quality of life, tailored information, and shared decision making were expressed in different ways by the participants. Such a patient approach, also called a patient-centered approach, may facilitate integration, improve care, and avoid futile treatment.^25–27^ However, that would infer interprofessional collaboration^28^ that at present is considered insufficient, at least by the oncology nurses. In addition, several participants questioned the clinical practice of providers in other professions while they viewed their own practice more positively. The identified positive self-view is explained by attribution theory as typical behavior to maintain confidence in ourselves.^29^ Another component of this theory is the tendency to explain challenges, difficulties, or failures externally also to maintain a positive self-view. This would fit with our results of HCPs' description of others and organizational factors as barriers to optimal patient care. Furthermore, it could also explain the low level of learning needs reported among the participants. Awareness of the tendency to make attributions of one's own and others' behavior might be important to acknowledge a need for enhancement of one's own skills. It could also be essential to consider when planning interventions.^30,31^

Second, organizational challenges, such as time pressure, and the lack of routines for referrals and collaboration, were perceived to influence patient care. Physicians especially emphasized that sufficient time in consultations was necessary when addressing difficult topics. Acquisition of skills making the consultations more efficient without compromising quality might be an effective action.^32,33^ However, the HCPs did not mention how they could contribute to making the consultations more efficient themselves, but presented this as a leadership challenge. The external attribution already described seems to apply here as well. The importance of external attributions and lack of self-perceived learning needs among HCPs have received little attention in the literature. One might assume that these factors work as barriers to change, as well as to interprofessional collaborations.^16^

Third, closely linked to the organizational challenges were a number of critical appraisals to colleagues in other professions and positions. In general, an insufficient level of communication and collaboration upward in the hierarchy appeared to be a challenge, which is also reported elsewhere.^34^ Nurses and residents were more aware of this than the oncologists and palliative care physicians. Some nurses described difficulties in addressing the physicians due to fear of rejection. However, well-functioning collaboration can improve patient outcomes and job satisfaction.^34,35^ Knowledge of each other and available arenas to meet are two organizational-related factors that could be facilitators.^16,20^

However, the current view on self and other HCPs seems to be a barrier that needs to be taken into account. For example, the oncologists neither experienced a great need for improved collaboration with palliative care services nor identified a need for earlier referrals of “their” patients. A need for palliative measures earlier in the disease trajectory was, however, identified by all the other groups. One of the palliative care physicians called it a need for an expansion of “the palliative mindset” with less focus solely on tumor-directed treatment.

The different cultures these professions have been socialized into during their education, clinical practice, and through public perceptions might also influence attitudes and behavior toward each other and the patients.^36,37^ Although physicians for centuries have been focusing on cure, nurses have been taught to focus on care.^38^ Care and quality of life have also been the main focuses in palliative medicine.^39^ These differences are by no means conflicting, but might still point out why a shared mental model is difficult to achieve. We identified what could be examples of this in the interviews, specifically in the area of tumor-directed treatment focus that was described by nurses, residents, and palliative care physicians as prevailing among the oncologists. Our, and others, interpretation is that there are both historical and cultural gaps between the oncological and the palliative care mindsets, which may play an important part in how the HCPs view each other and interact.^40–42^ Possibly this has an impact on the care provided as well: in delayed referral to palliative care, limited collaboration, and decreased patient-centered care as potential consequences. Still, and as illustrated in the first theme, everyone agrees on the main goal: to provide best possible care for the patients. Interventions contributing to improved collaboration to reach this overall goal may counteract culture-biased attitudes and behavior and improve interprofessional interactions.^43^

### Implications

The results of the thematic analysis provided us with important information for the development and implementation of the PALLiON intervention. The results might also have implications for improving collaboration and integration in advanced cancer care in general.

In particular, the novel findings of favorable self-assessment, external attribution of barriers, and low self-perceived learning needs expressed by the participants are important to consider. An intervention aiming for decreased fragmentation with specific referral practices, improved but not unwarranted continuity, and formalized arenas for interprofessional collaboration could be one measure. These elements are now being tested in the PALLiON study. Although the HCPs in this study did not report pressing learning needs, they highlighted some challenges in patient care, such as communicating with young patients with small children, who were included in the intervention as well. Trying to influence the philosophy of care, moving toward a combined focus upon both tumor-directed treatment and patient-centered care is of crucial importance. Resistance among HCPs to such interventions should be anticipated,^41,42^ but could be counteracted by facilitation of finding common ground and shared goals.

### Strengths and limitations

This study is based on interviews with HCPs from a single Norwegian center, although from two separately located campuses and from different sections. Although the findings seem generic and recognizable, we do not know how a potential single-center bias or the sizes of the sections have affected the results. As in all such studies, the most interested and motivated individuals participated and we do not know how this bias might have affected our data.

## Conclusion

Our findings indicate a patient-centered ideology across professional groups, but limited consensus on how it functions in daily clinical practice. Currently, improving collaboration to achieve integrated care appears to be challenging especially due to HCPs' positive self-view and externalization of challenges in patient care. Resistance to changing their own behavior combined with a perceived need for organizational changes might be two essential obstacles to improvement that are reinforced by cultural differences and perceptions. Interventions targeting both HCP-related and organizational factors are needed. Particularly important are measures aimed at reducing fragmentation and improving collaboration in care, such as creating formal and informal arenas to meet other HCPs, educational initiatives to improve skills and communication, and establishing routines around referral practices and continuity in care providers.

## Abbreviation Used

HCPs: health care providers

## References

[1] Organisation for Economic Cooperation and Development (OECD): Health at a Glance 2015: OECD Indicators (Summary in Norwegian). Paris: OECD Publishing, 2015

[2] Lee J, Johnson SR: “Fragmented” cancer care—System is “chaotic, costly,” IOM report says. Modern Healthcare 2013;43:8–9 24195156

[3] Allen D, Badro V, Denyer-Willis L, et al.: Fragmented care and whole-person illness: Decision-making for people with chronic end-stage kidney disease. J Chronic Illness 2015;11:44–55 10.1177/1742395314562974 25475415

[4] Iversen H, Bjertnaes Ø: Pakkeforløp for kreft—Resultater fra spørreskjemaundersøkelser i befolkningen og blant pasienter [Cancer patient pathways: Results from surveys among the general population and patients]. Folkehelseinstituttet. https://fhi.brage.unit.no/fhi-xmlui/bitstream/handle/11250/2486269/K_PasOpp_2016_Pakkeforl%25C3%25B8p%2Bfor%2Bkreft.pdf?sequence=2&isAllowed=y. 2016. (Last accessed 52020)

[5] Hannisdal E, Arianson H, Braut GS, et al.: A risk analysis of cancer care in Norway: The top 16 patient safety hazards. Jt Comm J Qual Saf 2013;39:511-AP1 10.1016/s1553-7250(13)39067-9 24294679

[6] Årsmelding Pasient og Brukerombudet 2019. Pasient og Brukerombudet. https://helsenorge.no/pobo-seksjon/Documents/Årsmelding%202019.pdf. [Annual report 2019, the Health and Social Services Ombudsmen. https://helsenorge.no/pobo-seksjon/Documents/Annual%20Report%202019.pdf] 2019. (Last accessed 52020)

[7] Nasjonalt handlingsprogram for palliasjon i kreftomsorgen. [Norwegian Directory of Health. Nasjonale handlingsprogrammer for kreft—national treatment guidelines for cancer.] https://www.helsedirektoratet.no/retningslinjer/palliasjon-i-kreftomsorgen-handlingsprogram: Helsedirektoratet. 2015. (Last accessed 52020)

[8] Norwegian Directory of Health. Leve med kreft—living with cancer, National Cancer Strategy 2018–2022. https://www.regjeringen.no/contentassets/266bf1eec38940888a589ec86d79da20/regjeringens_kreftstrategi_180418.pdf. 2018. (Last accessed 52020)

[9] På liv og død: Palliasjon til alvorlig syke og døende. [Official Norwegian Reports. On Life and death—palliative care for seriously ill and dying patients.] https://www.regjeringen.no/no/dokumenter/nou-2017-16/id2582548/Oslo. 2017. (Last accessed 52020)

[10] Lindemann K, Liland H, Kloppen B, et al.: When to stop futile treatment towards end of life in gynaecological cancer patients: A population-based study in oslo county, Norway. Conference Paper: ESGO, 2019

[11] Wentlandt K, Krzyzanowska MK, Swami N, et al.: Referral practices of oncologists to specialized palliative care. JCO 2012;30:4380–4386 10.1200/JCO.2012.44.0248 23109708

[12] Zimmermann C, Swami N, Krzyzanowska M, et al.: Early palliative care for patients with advanced cancer: A cluster-randomised controlled trial. Lancet 2014;383:1721–1730 24559581 10.1016/S0140-6736(13)62416-2

[13] Vanbutsele G, Pardon K, Van Belle S, et al.: Effect of early and systematic integration of palliative care in patients with advanced cancer: A randomised controlled trial. Lancet Oncol 2018;19:394–404 29402701 10.1016/S1470-2045(18)30060-3

[14] Von Roenn JH, Voltz R, Serrie A: Barriers and approaches to the successful integration of palliative care and oncology practice. J Natl Compr Canc NE 2013;11(Suppl. 1):11–16 10.6004/jnccn.2013.0209 23520181

[15] Marcia G, Ronit E, Betty F, et al.: Current status of palliative care—Clinical implementation, education, and research. CA Cancer J Clin 2009;59:327–335 19729681 10.3322/caac.20032 PMC2929964

[16] Bennardi M, Diviani N, Gamondi C, et al.: Palliative care utilization in oncology and hemato-oncology: A systematic review of cognitive barriers and facilitators from the perspective of healthcare professionals, adult patients, and their families. BMC Palliat Care 2020;19:1–17 32284064 10.1186/s12904-020-00556-7 PMC7155286

[17] Moore PM, Rivera Mercado S, et al.: Communication skills training for healthcare professionals working with people who have cancer. Cochrane Database Syst Rev 2013;2013:CD003751 10.1002/14651858.CD003751.pub3 PMC6457800 23543521

[18] Bernabeo E, Holmboe ES: Patients, providers, and systems need to acquire a specific set of competencies to achieve truly patient-centered care. Health Affairs 2013;32:250–258 23381517 10.1377/hlthaff.2012.1120

[19] Kruser TJ, Kruser JM, Gross J, et al.: Barriers to early integration of palliative care: A qualitative analysis of medical oncologist attitudes and practice patterns. ASCO 2018;36:e22191

[20] Earp M, Sinnarajah A, Kerba M, et al.: Opportunity is the greatest barrier to providing palliative care to advanced colorectal cancer patients: A survey of oncology clinicians. Current Oncol 2018;25:e480 10.3747/co.25.4021 PMC6209543 30464700

[21] Dhollander N, De Vleminck A, Robijn L, et al.: Barriers for the early integration of palliative home care into the disease trajectory of advanced cancer patients: A focus group study with palliative home care teams. J Pain Symptom Manage 2018;56:e25–e26 10.1111/ecc.13024 30784150

[22] Braun V, Clarke V: Using thematic analysis in psychology. Qual Res Psychol 2006;3:77–101

[23] DeSantis L, Ugarriza DN: The concept of theme as used in qualitative nursing research. West J Nurs Res 2000;22:351–372 10804897 10.1177/019394590002200308

[24] Institute of Medicine (IOM): Crossing the Quality Chasm: A New Health System for the 21st Century. Washington, DC: National Academies Press, 2001 25057539

[25] Fallowfield LJ, Jenkins VA, Beveridge H: Truth may hurt but deceit hurts more: Communication in palliative care. Pall Med 2002;16:297–303 10.1191/0269216302pm575oa 12132542

[26] Gattellari M, Voigt KJ, Butow PN, et al.: When the treatment goal is not cure: Are cancer patients equipped to make informed decisions? JCO 2002;20:503–513 10.1200/JCO.2002.20.2.503 11786580

[27] Fallowfield L, Lipkin M, Hall A: Teaching senior oncologists communication skills: Results from phase I of a comprehensive longitudinal program in the United Kingdom. JCO 1998;16:1961–1968 10.1200/JCO.1998.16.5.1961 9586916

[28] Leutz WN: Five laws for integrating medical and social services: Lessons from the United States and the United Kingdom. Milbank Quart 1999;77:77–110 10.1111/1468-0009.00125 PMC2751110 10197028

[29] Zuckerman MJ: Attribution of success and failure revisited, or: The motivational bias is alive and well in attribution theory. J Pers 1979;47:245–287

[30] Sommaruga M, Casu G, Giaquinto F, et al.: Self-perceived provision of patient centered care by healthcare professionals: The role of emotional intelligence and general self-efficacy. Pat Edu Couns 2017;100:974–980 10.1016/j.pec.2016.12.002 27986393

[31] Cooley C: Communication skills in palliative care. Prof Nurse 2000;15:603–605 11129941

[32] Frankel RM, Stein T: Getting the most out of the clinical encounter: The four habits model. Perm J 1999;3:79–88 11317576

[33] Stein T, Frankel R, Krupat E: Enhancing clinician communication skills in a large healthcare organization: A longitudinal case study. Pat Edu Couns 2005;58:4–12 10.1016/j.pec.2005.01.014 15950831

[34] Schmalenberg C, Kramer M: Nurse-physician relationships in hospitals: 20 000 nurses tell their story. Crit Care Nurse 2009;29:74–83 10.4037/ccn2009436 19182283

[35] Siedlecki S, Hixson E: Relationships between nurses and physicians matter. OJIN 2015;20:6 26882515

[36] Martinsen EH: Care for nurses only? Medicine and the perceiving eye. Health Care Anal 2011;19:15–27 10.1007/s10728-010-0161-9 PMC3037482 21136173

[37] Branch WT, Jr: The ethics of caring and medical education. Acad Med 2000;75:127–132 10.1097/00001888-200002000-00006 10693842

[38] Fox E: Predominance of the curative model of medical care: A residual problem. JAMA 1997;278:761–763 9286838

[39] Nyatanga B: The essential pillars of palliative care. Br J Community Nurs 2016;21:418 27479857 10.12968/bjcn.2016.21.8.418

[40] Gidwani R, Nevedal A, Patel M, et al.: The appropriate provision of primary versus specialist palliative care to cancer patients: Oncologists' perspectives. J Palliat Med 2017;20:395–403 10.1089/jpm.2016.0399 27997278

[41] Tartaglione EV, Vig EK, Reinke LF: Bridging the cultural divide between oncology and palliative care subspecialties: Clinicians' perceptions on team integration. Am J Hosp Palliat Care 2017;35:978–984 10.1177/1049909117747288 29258319

[42] Hall P: Interprofessional teamwork: Professional cultures as barriers. J Interprof Care 2005;19(Suppl. 1):188–196 10.1080/13561820500081745 16096155

[43] Gagliardi AR, Dobrow MJ, Wright FC: How can we improve cancer care? A review of interprofessional collaboration models and their use in clinical management. Surg Oncol 2011;20:146–154 10.1016/j.suronc.2011.06.004 21763127

